# Genetics and diagnostics of inherited retinal diseases in the era of whole genome sequencing

**DOI:** 10.1515/medgen-2024-2049

**Published:** 2025-02-12

**Authors:** Heidi Stöhr, Bernhard H. F. Weber

**Affiliations:** Institute of Human Genetics Institute of Human Genetics Franz-Josef-Strauß-Allee 11 93053 Regensburg Germany; Institute of Human Genetics Institute of Human Genetics Regensburg Germany

**Keywords:** inherited retinal disease, diagnostic yield, whole genome sequencing, structural variants, deep intronic variants

## Abstract

Inherited retinal diseases are clinically and genetically highly heterogeneous conditions with many phenotypic overlaps, syndromic presentations and atypical manifestations. This article is a narrative review that offers an overview of the technical advancements improving the accuracy and efficiency of molecular genetic diagnostics for hereditary disorders in clinical practice. It focuses particularly on the integration of whole genome sequencing (WGS) into routine diagnostics, critically evaluating its potential by discussing recent data from cohort studies conducted worldwide.

## Introduction

Inherited retinal diseases (IRDs) represent a broad group of degenerative eye disorders that can be classified into localized and generalized retinal/choroidal dystrophies, typically presenting with a progressive decline of visual function due to photoreceptor loss in either central or peripheral regions of the retina [19]. Additionally, some retinal diseases display a more stationary phenotype, involve functional impairment of ganglion cells, manifest with hereditary malformation of retinal blood vessels, or present with other ocular or extraocular symptoms that may also affect almost all organs and tissues of the human body, specifically the kidneys, ears, heart, and central nervous system [35]. Although most of the individual non-syndromic and syndromic IRD entities are very rare, collectively, they add up to a frequency of up to 1:1,300 in the general population, leading to an estimated 5.5 million people worldwide affected with some form of hereditary retinal disease [15].

**Figure 1:**
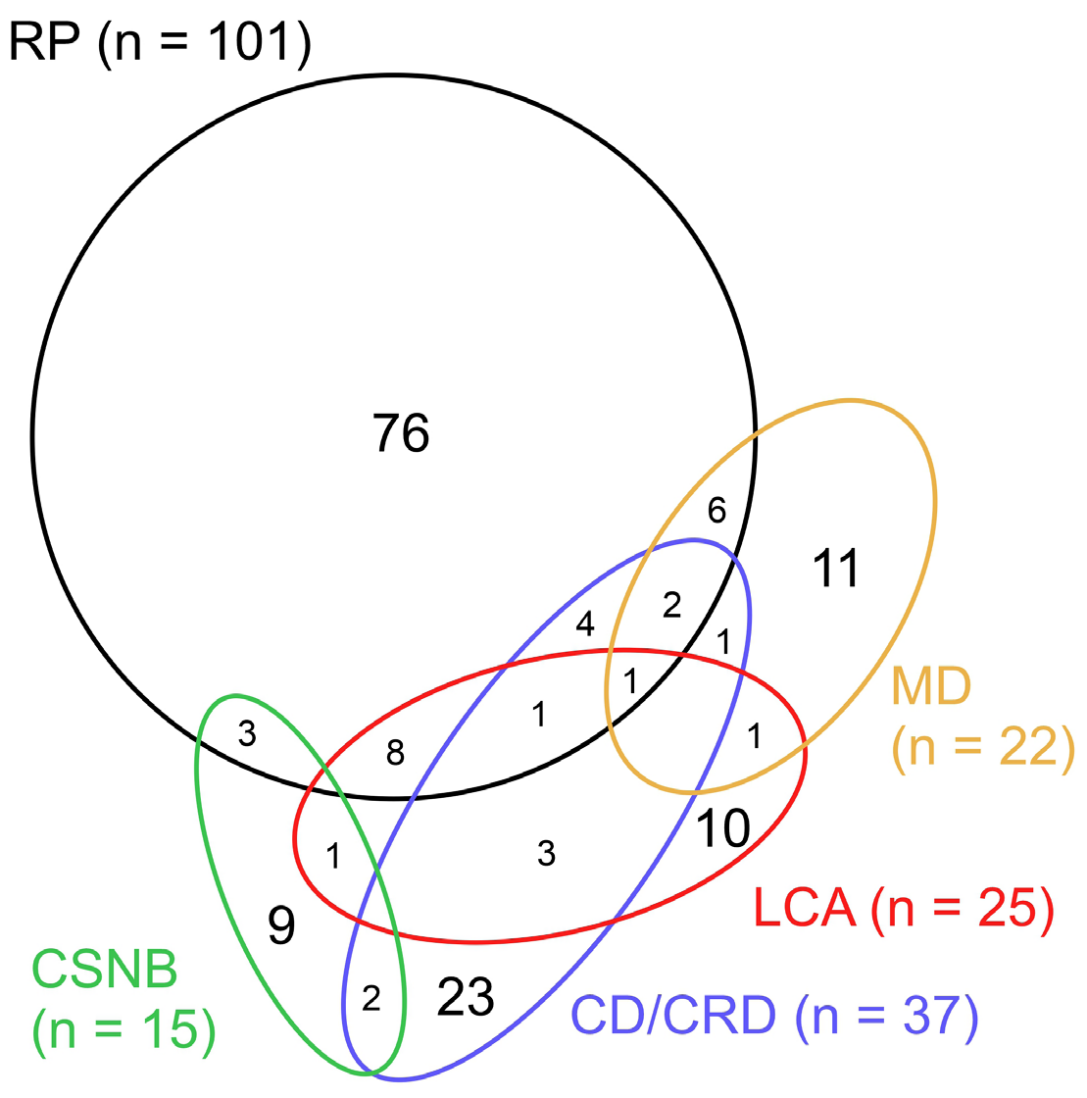
Venn diagram showing the number of distinct and overlapping genes associated with different forms of non-syndromic IRD (data taken from [2]). RP = retinitis pigmentosa, MD = macular dystrophy. LCA = Leber congenital amaurosis, CD/CRD = cone dystrophy/cone-rod dystrophy, CSNB = congenital stationary night blindness.

Several aspects of IRD make DNA diagnostics particularly relevant for this group of diseases. Firstly, since the identification of the first pathogenic variant in the rhodopsin gene by Dryja et al. in 1990, over 400 genes and gene loci have been recognized as causally linked to retinal disease [1, 2]. Secondly, the most common IRDs exhibit genetic heterogeneity, arising from mutations in numerous genes (**Figure 1**), each with distinct function. For instance, typical features of retinitis pigmentosa (RP) are associated with mutations in *PRPF31*, a ubiquitously expressed pre-mRNA processing factor essential for the formation of the spliceosome [38], and on the other side with mutations in *CNGA1*/*CNGB1* encoding rod photoreceptor specific subunits of the heterotetrameric photoreceptor cyclic nucleotide-gated (CNG) channel, which mediate the electrical response to light during phototransduction [36]. Thirdly, mutations in several disease genes, such as *AIPL1* or* CFAP418*, may express overlapping or distinct clinical manifestations, leading to a further significant genetic and phenotypic heterogeneity (**Figure 1**). While 25 % of RP genes may be involved in various other retinal phenotypes, this proportion increases in cone dystrophy/cone rod dystrophy (CD/CRD) (38 %), congenital stationary night blindness (CSNB) (40 %), macular dystrophies (MD) (50 %), and Leber congenital amaurosis (LCA) (60 %). In addition, retinal abnormality may be the first and sometimes the only persistent symptom of a systemic disorder for years (e. g. autosomal-recessive spastic paraplegia [5], own unpublished family), making it indispensable to establish an accurate diagnosis by DNA testing.

A key reason to define the individual gene defect for a given IRD condition is the availability of personalized therapies (e. g., Voretigene Neparvovec, a subretinal gene replacement therapy for patients with biallelic mutations in the retinoid isomerohydrolase RPE65 [32]), the number of which is expected to increase in the near future. Moreover, an early molecular diagnosis for patients with atypical clinical presentations of IRD or with symptoms on the mild end of the spectrum of a more severe disorder becomes particularly crucial when therapeutic or prophylactic interventions are available. A positive genetic test result for retinal diseases such as Refsum disease or gyrate atrophy, can have significant and concrete implications for dietary interventions to slow disease progression. Genetic testing can also assist in differential diagnosis, helping to avoid unnecessary further examinations or incorrect treatments (e. g., distinguishing between retinitis pigmentosa and posterior uveitis). Furthermore, it can help define an individual’s risk for complications (e. g., secondary macular neovascularization or macular edema), allow for a more accurate prediction of disease prognosis, and provide an estimate of disease risk for descendants.

## DNA testing in IRD in the pre-WGS era

As with almost all hereditary diseases, the molecular genetic diagnosis of IRD was a laborious and costly process before the introduction of efficient high-throughput methods for the parallel analysis of numerous genes. For many years, the exons and exon-intron boundaries of IRD-associated genes were analyzed using direct DNA Sanger sequencing. This usually was applied to single, often smaller genes (e. g., *RHO*,* PRPH2*) or a few genes responsible for a specific IRD subtype (e. g. *RP2* and *RPGR* for X-chromosomal RP) [4]. Prescreening techniques such as single-strand conformation analysis, denaturing high-performance liquid chromatography and genotyping microarrays (arrayed primer extension analysis, APEX), developed to tackle the challenges of genetic heterogeneity and large-sized genes (e. g. *ABCA4*), were limited by their reduced sensitivity and specificity. Nevertheless, we and others determined diagnostic rates of 58 % [30] and up to 78 % [17] using these prescreening tools, which can be attributed to clinically and ethnically uniform study populations (e. g. European patients with Stargardt disease). The introduction of high-density resequencing microarrays for DNA testing marked a preliminary step towards the implementation of genomic strategies in IRD diagnostics facilitated by next-generation sequencing (NGS) [23, 24]. In the past decade and until today, targeted multigene panel-based sequencing, virtual gene panel or phenotype-based assessment of whole exome sequencing (WES) data have become standard practice in IRD genetic diagnostics. This further enhanced the likelihood of identifying the genetic basis of the disease with diagnostic rates ranging between 55–75 %, the numbers estimated from reasonably sized IRD patient cohorts worldwide [18, 27, 28]. The variability in the solve rates largely depends on the IRD subtype. Causative variants that explain the etiology responsible for the disease manifestation are more likely to be identified in patients with a distinct clinical IRD phenotype, as in X-linked juvenile retinoschisis. In contrast, other subtypes, like macular dystrophies, are characterized by diverse and overlapping clinical presentations, a high level of genetic heterogeneity and multifactorial pathogenesis leading to reduced diagnostic rates. Of note, diagnostic success rates may also be compromised by complexities involved in differential diagnosis of retinal disease. Specifically, diagnosing non-hereditary conditions that mimic retinal dystrophies, including those related to toxicity, inflammation, or tumors, can be particularly challenging.

**Figure 2:**
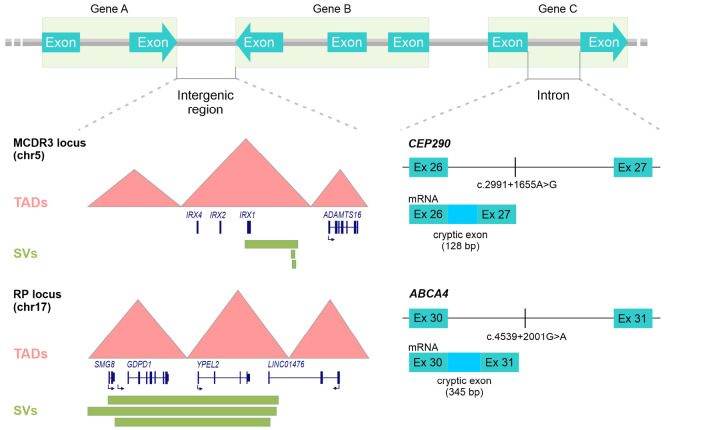
Schematic illustration of a selection of known IRD-causing non-coding variants in intergenic regulatory or intronic regions. For better visualization the exon-intron structures of three hypothetical genes highlighted by green boxes are shown on top. Below on the left, intergenic regions representing the MCDR3 and RP17 loci are delineated with TAD models plotted according to Hi-C maps [7, 37] in red, exon-intron structure of genes located in the TADs shown in blue and overlapping disease-associated structural variants (duplications or triplications) shown by green bars [7, 9, 34, 37]. Below on the right, intronic regions of the *CEP290* and *ABCA4* genes are depicted, each showing a deep intronic disease-associated sequence change leading to the insertion of a cryptic exon of different size into the respective mRNA [10, 33].

## Missing heritability in IRD: deep intronic splice and regulatory variants

RT-PCR analysis of the entire *CEP290* mRNA isolated from a lymphoblastoid cell line of an LCA patient revealed an aberrant splice product with an inserted cryptic exon due to a c.2991+1655A>G sequence change, which was the first deep intronic IRD-causing mutation identified and the most common causative variant in the *CEP290* gene (**Figure 2**) [10]. Over the years, several targeted whole-gene screening methods have been applied to search for other splice altering variants that might explain the missing heritability in IRD and successfully identified deep-intronic splice mutations in various IRD genes (e. g. *PRPF31* [29], *USH2A [14]*). The most notable efforts were made to detect intronic variants in the *ABCA4* gene that were presumed to have escaped identification during conventional diagnostic screening. In large cohorts, single molecule molecular inversion probes (smMIPs)-based sequencing of the entire *ABCA4* gene locus repeatedly contributed to genetically solve retinopathy cases by identifying novel deep intronic splice variants (**Figure 2**) [20].

Extensive research over the years have led to the discovery of sequence changes that are likely involved in regulating retinal gene expression as the underlying cause of rare subtypes of autosomal-dominant macular dystrophy and retinitis pigmentosa. For example, North Carolina macular dystrophy (NCMD) has been linked to non-coding single-nucleotide variants (SNVs) within DNase I hypersensitive sites upstream of the *PRDM13* gene and several independent duplication events encompassing this site on chromosome 6 (MCDR1) or another DNase I hypersensitive site near the *IRX1* locus on chromosome 5 (MCDR3) *[9, 34]* (Figure 2). NCMD-linked sequence changes in these putative cis-regulatory elements are probably dysregulating the transcription factor-encoding genes* IRX1* and* PRDM13* which then leads to abnormal retinogenesis [37]. Cis-regulatory enhancers with binding sites for retinal transcription factors are also affected by complex structural variants (SVs) in a genomic region on the long arm of chromosome 17 causing autosomal-dominant RP (RP17) (**Figure 2**) [7]. Functional investigations on the disease mechanism underlying this type of RP suggest that SVs disrupt topologically associating domains (TADs) and create neo-TADs enabling ectopic retinal enhancer-gene interactions that lead to abnormal retinal gene expression (notably of the *GDPD1* gene) [7]. Additionally, it is worth noting that complex interactions among known retinal disease-causing mutations in various genes have been reported. For instance, trans modifier genes like *ROM1* and *PRPH2* may influence the effects of causative *ABCA4* variants associated with Stargardt disease [41].

## Whole genome sequencing in IRD diagnostics

Several studies published in the last eight years exemplify the increasing use of WGS in unraveling the underlying causes of IRD. The overall diagnostic rates vary significantly among individual cohorts and range from a few percent to more than 50 % (**Table 1**). This wide spread of solve rates can mainly be attributed to the substantial differences in the inclusion criteria for cases and thus the composition of the study group. The extent of prescreening performed in patients and the proportion of carriers with known mono-allelic pathogenic variants in autosomal-recessive IRD genes included, is often unclear. Additional factors contributing to differences in solve rates could be due to variations in biostatistical methods used and differences in how the causal pathogenicity of genetic results is defined. Finally, important confounding factors may include discrepancies in the reliability of the underlying ophthalmologic diagnosis, such as differences in ophthalmologist expertise and the level of interdisciplinary collaboration.

One of the earliest reports on mutation-detection via WGS was published by Ellingford and colleagues [11], who observed a diagnostic rate of 52 % in their small cohort of 46 IRD patients (24/46). All patients had previously undergone targeted NGS analysis of 105 IRD genes and a molecular diagnosis had already been established in 13 of the 46 individuals and was confirmed by WGS. The remaining 33 patients included 11 carriers of a mono-allelic pathogenic variant in one of the IRD genes tested. WGS identified additional clinically relevant variants accounting for disease presentation in 11 of the 33 patients (33 %), eight of which had been known carriers of single heterozygous variants. In another study from the UK, Carss et al. identified the molecular cause in 345 of their 650 IRD patients by WGS (53 %) [8]. Forty-five of the patients had been prescreened by WES and most of the remaining individuals by different prescreening methods such as targeted Sanger sequencing, APEX, and NGS-based gene panels. A mix of genetic pretests had also been performed in 409 individuals from 108 IRD families recruited from Mexico, Pakistan and the USA [3]. WGS detected causative variants in 61 out of the 108 pedigrees (57 %). In another study with 100 IRD patients from diverse ethnic and geographic backgrounds (Netherlands, Israel, Ireland), 24 cases were solved by WGS (24 %) [12]. About half of the cohort each had been prescreened by either targeted capture-sequencing or WES which had identified mono-allelic pathogenic variants in known IRD genes in 56 individuals. Of interest, WGS detected a second disease-causing variant in only nine of the 56 carriers of single heterozygous variants. It was speculated that the employed prioritization strategy may have filtered out causative variants (e. g. hypomorphic alleles, regulatory variants) or that some variants may have been missed due to base calling errors [12]. A smaller Italian cohort of 33 patients with suspected autosomal-recessive IRD contained an even higher proportion of carriers of mono-allelic causative variants (30/33) [42]. The results of WGS analysis showed that of the 11 cases with an established molecular diagnosis, 10 had been known to carry single heterozygous variants. In a Chinese cohort encompassing 271 IRD patients for which a molecular diagnosis was not obtained by targeted gene panel testing, WGS revealed IRD-associated variants in 34 individuals (13 %) [21]. In 13 of the 34 patients, a mono-allelic disease-causing variant had already been detected by previous DNA testing. Oh et al. performed WGS in 33 unsolved Korean IRD patients prescreened by WES or targeted gene panel analysis [26]. They established a molecular diagnosis in five of them (16 %), three had been known to carry single heterozygous variants. In a Swiss study, WGS analysis led to a molecular diagnosis in 19 of 66 patients with IRD and related eye disorders unsolved following WES (29 %) [22]. As part of the multicenter study “Bavarian Genomes Network for Rare Disorders”, we have performed WGS in 65 non-related IRD families comprising 143 individuals with no clear result after prescreening with traditional DNA diagnostics tools. Preliminary data revealed disease causation in 13 of the 65 IRD families (20 %). The most comprehensive data on the utilization of diagnostic genome sequencing in patients with IRD has so far been published by another German group [39]. Prospective WGS was performed in 1,000 individuals from 968 families of which 206 had previously undergone first-tier genetic testing without discovering the cause of the disease by using different techniques. A definite genetic diagnosis was established in 57 % of all the cases (574 / 968). An approximately 10 % lower diagnostic yield (46 %) was achieved for the prescreened subcohort (94 / 206).

**Table 1:** Short-read WGS-based diagnostics in IRD cohorts

Study	YOP	Country	Index patient	pre-screen	Type (no. if known)	Solved cases (diagnostic yield %)		SV	non-coding variant	exp. assay	pathogenicity confirmed
						**total**	**mono-allelic in pre-screen**				
[11]	2016	UK	33	all	TGP	11 / 33 (30)	8 / 11 (72)	5	-	no	-
[8]	2017	UK	650	most	Sanger, APEX, TGP, WES (45)	345 / 650 (53)	n. s.	29	3	no	-
[3]	2021	US	108	all	Sanger, APEX, TGP, WES (31)	61 / 108 (57)	n. s.	5	-	no	-
[12]	2021	IR, IL, NL	100	all	TGP (46), WES (54)	24 / 100 (24)	9 / 24 (38)	2	13	mini / midi-gene assay	8 / 13
[15]	2023	US	311	all	TGP, WES	6 / 311 (2)	n. s.	-	4	minigene assay	2 / 2
[39]	2023	US	755	all	TGP, WES	16 / 755 (2)	n. s.	16	-	no	-
[20]	2024	CN	271	all	TGP	34 / 271 (13)	13 / 34 (38)	22	5	minigene assay	5 / 5
[21]	2024	CH	66	all	WES	19 / 66 (29)	n.s.	4	1	no	-
[25]	2024	KR	32	all	TGP, WES	5 / 32 (16)	3 / 5 (60)	2	1	no	-
[38]	2024	DE	968	206	Sanger (69), TGP (137)	574 / 968 (57) [94 / 206 (46)]	n. s.	71	63	RNA-seq	2 / 31
[40]	2024	IT	33	all	APEX (1), TGP (32)	11 / 33 (33)	10 / 11 (91)	3	4	RT-PCR in lymphocytes	2 / 2
Wenzel et al.	unpublished	DE	65	all	TGP (61), WES (4)	13 / 65 (20)	0 / 13	6	5	in progress	-

YoP, year of publication; y, yes; APEX, arrayed primer extension; TGP, targeted gene panel; WES, whole exome sequencing; SV, structural variant; n.s., not specified; RT-PCR, reverse transcription polymerase chain reaction

Two WGS studies performed at the Baylor College of Medicine (USA) obtained diagnostic yields of only approximately 2 % each (**Table 1**). The reports, however, focus on findings of either structural variants [40] or deep intronic variants [16] in subgroups of a large cohort of 6,532 IRD patients making a comparison of the results of these investigations with the numbers generated by other groups difficult (**Table 1**).

The ability to capture non-coding variants and to reliably detect structural variants including CNVs affecting less than three exons, complex or copy-neutral genomic rearrangements (e. g. inversions and translocations) is a major advantage of WGS over targeted NGS-based gene panel analysis or exome sequencing. Findings of structural variants among the total number of causative variants in IRD range from 0,25 % to 7 % [3, 8, 39, 40]. In seven articles on WGS in IRD reviewed herein as well as in our own unpublished data, the count of structural variants is higher than that of non-coding single nucleotide variants/small insertions and deletions (**Table 1**). Maggi et al. [22] suggested that WGS diagnosed 3–6 times more of their patients by the detection of structural variants than intronic or regulatory short variants.

Most of the structural variants identified by the different WGS-based studies represent deletion or duplication events affecting one, more or all exons of an IRD gene, rarely do they impact neighboring genes. Consistent with this observation, of the 71 unique structural variants described in the study by Weisschuh et al. [39], only four occurred exclusively in non-coding genomic regions. It is often argued that improved algorithms to find copy number variants during WES or targeted gene-panel analysis should be suited to detect most of the genomic abnormalities. The high accuracy with which WGS is able to identify this type of mutation and the advantages of precise breakpoint identification of genomic rearrangements even in regions characterized by repetitive elements, however, should not be overlooked [25, 40]. In addition, the implementation of long-read WGS has been shown to be valuable in identifying hidden pathogenic structural variants in IRD, such as retrotransposon insertions [13].

Since most of the genes involved in IRD are exclusively expressed in the non-accessible retina/RPE/choroid, *in vitro* functional assays are often used to assess the impact of variants at the mRNA level [31]. Five of the 11 WGS studies provided additional data on the effect of a total of 52 variants in the non-coding genome or at non-canonical splice-sites either by conducting mini/midigene assays, RT-PCR analysis or RNA-sequencing on patients’ blood lymphocytes (**Table 1**). Aberrant splicing events were identified for 19 (36 %) of the variations tested. Since gene expression and mRNA splicing are often tissue- and/or cell-specific processes, the *in vitro* validation of functional variants is challenging. Patient-derived induced pluripotent stem cells (iPSCs) differentiated *in vitro* to form retinal organoids can potentially resolve these limitations. In a pilot study, a combination of WGS and RNA-Seq, in addition to iPSC-derived retinal organoid transcriptome analysis were used to uncover tissue-specific splicing patterns resulting from non-coding variants [6]. Utilizing this strategy, a novel pathogenic deep intronic variant in the *CNGB3* gene that activates cryptic splice sites was found in a family with cone dysfunction.

## Conclusion and Outlook

The implementation of NGS technology into IRD research and routine diagnostics has expanded our knowledge of the genetic causes underlying IRDs and has enabled rapid molecular diagnosis for a large fraction of IRD patients paving the way for future personalized therapies. WGS has proven to be highly effective for the reliable detection and precise characterization of structural variants, including complex cases. The additional diagnostic value of WGS compared to conventional NGS methods, however, appears limited. Genomic diagnostics naturally unveils a high number of non-coding variants per patient, many of which have a low allele frequency in the general population and, if located within or in close proximity to an IRD gene locus, could be involved in IRD disease pathogenesis. The development of computational and feasible experimental strategies to evaluate the pathogenic effects of non-coding, putative IRD-causing variants are essential and pose a major task for the future. Another field of intense development is the introduction of artificial intelligence (AI) in ophthalmology. Many examples show that AI can support more accurate ophthalmologic phenotyping and differential diagnosis of retinal dystrophies, help establish more precise genotype-phenotype correlations, and assist in optimizing the analysis of the vast amounts of data generated by WGS.
